# Prevention and Rehabilitation After Heart Transplantation: A Clinical Consensus Statement of the European Association of Preventive Cardiology, Heart Failure Association of the ESC, and the European Cardio Thoracic Transplant Association, a Section of ESOT

**DOI:** 10.3389/ti.2024.13191

**Published:** 2024-06-19

**Authors:** Maria Simonenko, Dominique Hansen, Josef Niebauer, Maurizio Volterrani, Stamatis Adamopoulos, Cristiano Amarelli, Marco Ambrosetti, Stefan D. Anker, Antonio Bayes-Genis, Tuvia Ben Gal, T. Scott Bowen, Francesco Cacciatore, Giuseppe Caminiti, Elena Cavarretta, Ovidiu Chioncel, Andrew J. S. Coats, Alain Cohen-Solal, Flavio D’Ascenzi, Carmen de Pablo Zarzosa, Andreas B. Gevaert, Finn Gustafsson, Hareld Kemps, Loreena Hill, Tiny Jaarsma, Ewa Jankowska, Emer Joyce, Nicolle Krankel, Mitja Lainscak, Lars H. Lund, Brenda Moura, Kari Nytrøen, Elena Osto, Massimo Piepoli, Luciano Potena, Amina Rakisheva, Giuseppe Rosano, Gianluigi Savarese, Petar M. Seferovic, David R. Thompson, Thomas Thum, Emeline M. Van Craenenbroeck

**Affiliations:** ^1^ Cardiopulmonary Exercise Test Research Department, Heart Transplantation Outpatient Department, V. A. Almazov National Medical Research Centre, St. Petersburg, Russia; ^2^ REVAL and BIOMED Rehabilitation Research Center, Hasselt University, Hasselt, Belgium; ^3^ Heart Centre Hasselt, Jessa Hospital, Hasselt, Belgium; ^4^ University Institute of Sports Medicine, Prevention and Rehabilitation, Paracelsus Medical University, Salzburg, Austria; ^5^ Cardio-Pulmonary Department, IRCCS San Raffaele Roma, Rome, Italy; ^6^ Heart Failure and Heart Transplantation Unit, Onassis Cardiac Surgery Center, Athens, Greece; ^7^ Department of Cardiac Surgery and Transplants, Monaldi Hospital, Azienda dei Colli, Naples, Italy; ^8^ Cardiovascular Rehabilitation Unit, ASST Crema, Santa Marta Hospital, Rivolta D’Adda, Italy; ^9^ Department of Cardiology (CVK), Berlin Institute of Health Center for Regenerative Therapies (BCRT), German Centre for Cardiovascular Research (DZHK) Partner Site Berlin, Charité Universitätsmedizin Berlin, Berlin, Germany; ^10^ University Hospital Germans Trias and Pujol de Badalona, Badalona, Spain; ^11^ Heart Failure Unit, Cardiology Department, Rabin Medical Center, Petah Tikva and Sackler, Faculty of Medicine, Tel Aviv University, Tel Aviv, Israel; ^12^ School of Biomedical Sciences, Faculty of Biological Sciences, University of Leeds, Leeds, United Kingdom; ^13^ Department of Translational Medicine, University of Naples “Federico II”, Naples, Italy; ^14^ Department of Medical-Surgical Sciences and Biotechnologies, Sapienza University of Rome, Rome, Italy; ^15^ Mediterranea Cardiocentro, Naples, Italy; ^16^ Emergency Institute for Cardiovascular Diseases “Prof. C. C. Iliescu”, Bucharest, Romania; ^17^ University of Medicine Carol Davila, Bucharest, Romania; ^18^ Heart Research Institute, Sydney, NSW, Australia; ^19^ Cardiology Department, University of Paris, INSERM UMRS-942, Hopital Lariboisiere, AP-HP, Paris, France; ^20^ Division of Cardiology, Department of Medical Biotechnologies, University of Siena, Siena, Italy; ^21^ Department of Cardiology, Ramón y Cajal University Hospital, Madrid, Spain; ^22^ Research Group Cardiovascular Diseases, Genetics, Pharmacology and Physiopathology of Heart, Blood Vessels and Skeleton (GENCOR) Department, University of Antwerp, Antwerp, Belgium; ^23^ Department of Cardiology, Antwerp University Hospital, Edegem, Belgium; ^24^ Department of Cardiology, Rigshospitalet, University of Copenhagen, Copenhagen, Denmark; ^25^ Department of Cardiology, Maxima Medical Centre, Eindhoven, Netherlands; ^26^ Department of Industrial Design, Eindhoven University of Technology, Eindhoven, Netherlands; ^27^ School of Nursing and Midwifery, Queen’s University Belfast, Belfast, United Kingdom; ^28^ Department of Health, Medicine and Caring Science, Linköping University, Linköping, Sweden; ^29^ Julius Center, University Medical Center Utrecht, Utrecht, Netherlands; ^30^ Department of Heart Diseases, Wroclaw Medical University, Wroclaw, Poland; ^31^ Department of Cardiology, Mater University Hospital, Dublin, Ireland; ^32^ School of Medicine, University College Dublin, Dublin, Ireland; ^33^ Universitätsmedizin Berlin Campus Benjamin Franklin Klinik für Kardiologie Charite, Berlin, Germany; ^34^ General Hospital Murska Sobota, Murska Sobota, Slovenia; ^35^ Department of Medicine, Karolinska Institutet and Heart and Vascular Theme, Karolinska University Hospital, Stockholm, Sweden; ^36^ Armed Forces Hospital, Porto, Portugal; ^37^ Centre for Health Technologies and Services Research, Faculty of Medicine of University of Porto, Porto, Portugal; ^38^ Department of Cardiology, Oslo University Hospital Rikshospitalet, Oslo, Norway; ^39^ Division of Physiology and Pathophysiology, Otto Loewi Research Center for Vascular Biology, Immunology and Inflammation, Medical University of Graz, Graz, Austria; ^40^ Vetsuisse Faculty, University of Zurich, Zurich, Switzerland; ^41^ Dipartimento Scienze Biomediche per la Salute, Universita’ Degli Studi di Milan, Milan, Italy; ^42^ Cardiologia Universitaria, IRCCS Policlinico San Donato, Milan, Italy; ^43^ University Hospital of Bologna, Bologna, Italy; ^44^ Department of Cardiology, Scientific Institution of Cardiology and Internal Diseases, Almaty, Kazakhstan; ^45^ Department of Cardiology, Kapshagai City Hospital, Almaty, Kazakhstan; ^46^ St. George’s Hospital NHS Trust University of London, London, United Kingdom; ^47^ Division of Cardiology, Department of Medicine, Karolinska Institutet, Stockholm, Sweden; ^48^ Faculty of Medicine and Heart Failure Center, University of Belgrade, Belgrade University Medical Center, Belgrade, Serbia; ^49^ Institute of Molecular and Translational Therapeutic Strategies (IMTTS), Hannover Medical School and Fraunhofer Institute for Toxicology and Experimental Research, Hannover, Germany

**Keywords:** diabetes, dyslipidaemia, exercise training, heart failure, heart transplantation

## Abstract

Little is known either about either physical activity patterns, or other lifestyle-related prevention measures in heart transplantation (HTx) recipients. The history of HTx started more than 50 years ago but there are still no guidelines or position papers highlighting the features of prevention and rehabilitation after HTx. The aims of this scientific statement are (i) to explain the importance of prevention and rehabilitation after HTx, and (ii) to promote the factors (modifiable/non-modifiable) that should be addressed after HTx to improve patients’ physical capacity, quality of life and survival. All HTx team members have their role to play in the care of these patients and multidisciplinary prevention and rehabilitation programmes designed for transplant recipients. HTx recipients are clearly not healthy disease-free subjects yet they also significantly differ from heart failure patients or those who are supported with mechanical circulatory support. Therefore, prevention and rehabilitation after HTx both need to be specifically tailored to this patient population and be multidisciplinary in nature. Prevention and rehabilitation programmes should be initiated early after HTx and continued during the entire post-transplant journey. This clinical consensus statement focuses on the importance and the characteristics of prevention and rehabilitation designed for HTx recipients.

## Introduction

Heart transplantation (HTx) continues to be the most optimal therapeutic option for selected patients with end-stage heart dis-ease of varying aetiologies, with more than 6,000 heart transplants performed annually worldwide [1].

The history of HTx started more than 50 years ago but only recently European guidelines and position papers have recognized the importance of prevention (including medication, nutrition, and exercise prescription) in cardiovascular disease. However, optimal prevention in HTx recipients remains to be formulated [2, 3].

The aims of this clinical consensus statement are (i) to point out the importance and content of prevention and rehabilitation after HTx, and (ii) to promote the (non-)modifiable risk factors that should be addressed after HTx to improve patients’ capabilities and functional capacity, quality of life and survival.

## Features of the Heart Transplant Population

Little is known about the physical activity (PA) patterns, among other prevention measures, among patients after HTx. Firstly, HTx recipients face total denervation of the transplanted heart, with a significant impact on their response to exercise and daily-life activities [1]. Secondly, life-long immunosuppression leads to a recip-ients’ immunocompromised status and atypical clinical symptoms and is also associated with numerous post-transplantation compli-cations. Hence, it would be incorrect to consider HTx recipients as individuals without any chronic heart disease anymore, and they are clinically absolutely different from chronic heart failure (CHF) patients or those who are supported by mechanical circulatory support (e.g., left ventricular assist device). Therefore, prevention and rehabilitation after HTx is very specific and requires a multidis-ciplinary approach, and such programmes should be initiated early after HTx and continued during the whole post-transplant journey.

## Risk Conditions After Heart Transplantation: The Need for Multidisciplinary Prevention and Rehabilitation

With the improved survival over time of patients with heart disease due the availability of better medical, surgical, and device-based therapies, the clinical characteristics of the HTx recipient popu-lation have also evolved. Recent changes in donated organ alloca-tion policies in the United States in 2018 [4–6] and in many other countries around the world [7, 8] have led to organ allocations going to sicker, device-dependent and more frail end-stage CHF patients. In the effort to maximize the positive impact of HTx on post-transplant functional capacity, exactly these patients are expected to experi-ence greater benefits from intensive rehabilitation programmes.

Actually, HTx is not a complete cure for CHF. Long-term survival of HTx patients remains limited and exercise capac-ity and health-related quality of life (HRQoL) of HTx recipients remains inferior to age-matched healthy people [9]. To improve the exercise/functional capacity and HRQoL of HTx recipients, mul-tidisciplinary cardiac rehabilitation (CR) is an integral component in most clinical HTx programmes [10]. Hence, multidisciplinary pre-vention and rehabilitation will benefit from including the different healthcare practitioners, such as the heart transplant cardiologist, endocrinologist, lipidologist, nutritionist, physiotherapist, physia-trist, psychiatrist/psychologist, and social workers. Exercise training (ET) leads to an improved exercise/functional capacity, which in turn facilitates many PAs of everyday life. Tailored programmes of rehabilitation and prevention may better avoid complications that otherwise would negatively impact the patients’ HRQoL. Therefore, preoperative risk factors such as frailty, sarcopenia, overweight and physical deconditioning should be assessed and addressed accordingly.

The holistic multidimensional prevention and treatment of dia-betes, overweight, dyslipidaemia, and psychological wellness should not be restricted to the immediate postoperative recovery period but should be reassessed and readdressed periodically to counter-act their potential negative clinical impact after HTx.

Exercise training might play an important role in counteract-ing some of the side-effects associated with immunosuppression, such as diabetes, dyslipidaemia and hypertension, as well as skele-tal muscle dysfunction and an increased risk for infections. ET has the potential to alleviate these side-effects to some degree [11], albeit systematic studies are lacking. A structured collection of patient-reported outcome measures using modern devices and software should be implemented to measure and tailor the man-agement and prevention of complications after HTx. Indeed, once a significant survival benefit has been reached, HTx profession-als and patients face a mindset from improving survival towards improvement in HRQoL.

Areas for intervention should include lifestyle changes, psy-chosocial support, in addition to targeting improved control of hypertension, dyslipidaemia, atherosclerosis, and cardiac allograft vasculopathy (CAV) [12].

## Prevention After Heart Transplantation

### Lifestyle Changes and Sex

Metabolic derangements such as dyslipidaemia and post-transplant diabetes mellitus (PTDM) are important risk factors for graft fail-ure, coronary events and mortality after organ transplantation [13]. Other lifestyle-related factors contributing to an increased cardio-vascular risk and functional disability include a reduced exercise capacity [14], and significant psychosocial issues (i.e., clinical depres-sion, mood disorders with negative affect, demoralization, and coping problems) [15]. Therefore, lifestyle modification should be an important part of the management of HTx patients. Key elements of such lifestyle management programmes include PA counselling and exercise prescription, nutritional counselling and healthy nutri-tion intervention, smoking cessation, psychosocial management, education and strategies to improve self-efficacy, self-care, and self-confidence. Other specific factors that should be taken into account include education and management of the side-effects of medical therapy (e.g., anti-rejection and antihypertensive drugs, pre-vention and management of infections), interventions and services to help promote a return to work and social rehabilitation [16], as well as counselling on sexual activities. In general, patients can resume their sexual activities after HTx, if they can perform mild to mod-erate levels of PA without symptoms (i.e., empirically 1 W/kg body weight, which is the required tolerance of the physical demand of sexual activity) [17]. Therefore, transplant patients may benefit from education, counselling and CR [17–19]. Around 50% of HTx recipients report a decreased sexual frequency and/or libido [17]. HTx specific concerns include having difficulty integrating the new organ into their “sense of self” (i.e., avoiding sexual activity out of concern to protect their heart, anxieties about assuming the sexual identity of the donor, and perceptions of sexual unattractiveness). CR can improve the exercise capacity of patients after HTx, which may also enhance sexual functioning [18–20].

### Psychosocial Support and Return to Work

The psychosocial impact of referral, waiting for, and receiving a HTx is profound and variable, with many patients feeling fearful, anxious, guilty and inadequate, with concerns about the impact on their family [21, 22]. Despite the benefits of receiving a HTx, recipients need continual psychosocial as well as medical support, based on the understanding of the many complex challenges that can confront them. Moreover, patients often feel restricted in their ability to return to a normal life, due to the need for regular medical check-ups and possible hospital admissions.

The main objective of HTx is to increase survival [23, 24], as well as to improve PA and HRQoL, allowing patients to return to their daily activities [8, 25, 26] including returning to paid work. However, the rate of successful return to work after HTx is low and varies between different countries [17, 27–31]. Despite a satisfactory objective and subjective functional status, some patients choose not to return to work. Age [32], previous work [17] and time out of work before transplantation [15, 27, 31, 32] are determining factors in most studies, while the impact of functional capacity and educational level appears to be controversial [30]. The type of work previously performed may also play a role, with a return to work being more common in white-collar workers [32]. Identifying factors that affect post-transplant patient’s motivation to return to work might help health professionals to adopt the best course of treatment and psychological support, in order to fulfill this goal. Nevertheless, a return to work should not be considered as the only manifestation or measure of a patient’s real psychosocial condition [33–35]. After a long period of illness and the prospect of death, post-transplant patients tend to attach great importance to factors other than work, for example, giving priority to relationships with family and friends, spirituality and free time [27].

On the other hand, HTx recipients make use of all coping strategies, with predominance on problem-focused strategies. The use of these active coping strategies encompass behaviours that may lead to greater adherence to treatment [36]. Psychologically prepared individuals use more active coping strategies, which high-lights the importance of psychological support during the referral and transplantation process [36]. Hence, life-long rehabilitation and psychosocial support should be provided to HTx patients after the surgery. PA is one beneficial factor to improve peak oxygen uptake (VO_2peak_) in heart transplanted recipients based on the results of cardiopulmonary exercise testing (CPET) [37–40]. Although the exercise/functional capacity after HTx will improve significantly, changes in the type of work will sometimes be necessary, adapting it to the new situation and retraining for a different occupation. Obviously, the patient’s financial need and the lack of public health insurance will increase the urgence to return to work.

There is also the impact of immunosuppressive (IS) drugs on the recipients’ mental health, which can lead to anxiety and depres-sion (which are strong predictors for poor medication compliance or increased hospitalization rates in transplant recipients) [41]. Com-mon symptoms after cardiac surgery include fatigue or loss of energy, changes in sleep pattern, alterations to appetite, which often “may be misinterpreted by healthcare providers, researchers or patients as mood-related,” precisely because they resemble the somatic symptoms of depression [42]. Studies have demonstrated that psychosocial factors, particularly coping style and social support, may be significant predictors of morbidity and mortality in patients awaiting HTx [43, 44] and in the intermediate term after successful HTx [34, 45, 46]. Furthermore, recent studies found an increasing dete-rioration of emotional wellbeing in the long-term life course after HTx [47]. Hence, the existence of depression limits the return to work [32, 48]. It has also been shown that the patient’s feeling of ill-ness or the subjective assessment of his or her capacity for work, which does not always coincide with the real situation, can facilitate or limit return to work [17, 25, 32, 49]. The presence of clinical complica-tions (e.g., rejection, infections, etc.), as well as the presence of comorbidities will limit the return even after the patients recover from the transplantation [30]. While pre-transplant depression does not impact outcomes, patients with post-transplant depression are more likely to experience a complicated course, suggesting the need for increased vigilance regarding depression in such patients [50]. Moreover, patients with post-transplant depression within the first year have a significantly higher 5-year mortality [49].

All these aspects are advised during CR to improve the rate of return to work after HTx. In addition to ET, the rehabilitation process should focus on the psychological and educational aspects of the patient, self-esteem improvement and self-management of the illness, in function of the motivation to return to work.

### Complications of Immunosuppression

There is a common misconception that post-HTx recipients’ func-tional/exercise capacity usually returns to normal spontaneously. But the need for IS therapy actually may impair such recovery. Some of these IS-related complications, such as dyslipidaemia or osteoporosis, could be prevented, and some, such as tremor and leukopenia, can be managed by modifying the doses of the IS agents. Other adverse effects require prescription of new therapies (to address infections, endocrine abnormalities, neurological complica-tions, nephropathy and arterial hypertension [AH]) [6, 51–53]. Guide-lines for addressing the complications of IS therapy include regular screening for adverse events, minimizing drug doses, drug substi-tution, and drug withdrawal if possible, as well as initiating targeted therapies for a specific complication [54, 55]. It is important to assess for contraindications and drug interactions when medically treating complications associated with immunosuppression [56].

The available IS agents vary with respect to their risk for inducing PTDM. Mycophenolate mofetil and azathioprine have not been shown to have a large impact on insulin action or glu-cose metabolism and so do not appear to have a major role in PTDM. There is however increasing evidence that the commonly used IS agents, particularly calcineurin inhibitors (CNIs; tacrolimus, cyclosporine) and inhibitors of the mammalian target of rapamycin (mTOR; sirolimus and everolimus), may contribute to PTDM [53]. A major modifiable risk factor for the development of PTDM is IS therapy, but risk versus benefit analysis is needed to balance the risk of developing PTDM versus the risk of rejection. However, there are reports that both glucose intolerance and dyslipidaemia, that occur predominantly with mTOR inhibitors, improve as the drug dose is reduced [54]. However, increasing the risk of allograft rejection or transplant dysfunction is not justified by trying to man-age glucose intolerance and dyslipidaemia after HTx. High-dose corticosteroids, often used as part of induction protocols in the immediate post-transplant period, have a much greater negative impact on insulin sensitivity than the chronic low-dose corticos-teroids that are commonly prescribed in the maintenance of many IS protocols [55].

Reduction of mycophenolate mofetil or everolimus initiation may be beneficial in HTx patients presenting with leukopenia or neu-tropenia and lead to modest, short-term renal function improve-ments [55]. Other causes of leukopenia should be excluded such as infectious (invasive pulmonary aspergillosis, cytomegalovirus) and haematological complications [56–60].

Cardiovascular events may occur at an increased rate even independently of the HTx recipients’ age, sex, and cause of CHF. After HTx, development of dyslipidaemia, atherosclero-sis and AH is also associated with the prescribed doses of immunosuppression [60]. Pharmacological treatment of dyslipi-daemia is however effective [56]. Moreover, uncontrolled severe hyperlipidaemia and severe hypertriglyceridaemia associated with everolimus treatment may occur [61, 62]. Effective blood pressure (BP) control with antihypertensives has been shown to enhance graft survival and reduce the risk of future cardiovascular events in HTx recipients [63].

Malignancy after HTx is one the leading causes of mortality in the long term after HTx. According to the International Society for Heart and Lung Transplantation (ISHLT) guidelines, annual check-ups were recommended to screen all HTx recipients for breast, colon and prostate cancer and to have a close skin cancer surveillance, including education on preventive measures [56, 59]. The oncogenic Epstein—Barr virus is common in immunocompromised patients and also a key pathogenic driver in many post-transplant lymphoproliferative disorders (PTLD) cases [59]. However, chronic immunosuppression should be minimized in heart recipients with low immunological risk as possible, particularly at high risk for malignancy [56].

Complications after HTx vary according to the time from surgery and the IS therapy used as well as progression of pre-existing conditions. Future research needs thus to focus on reaching an optimal and customized balance between efficacy and toxicities of IS strategies [64].

### Arterial Hypertension

Pre-transplant AH is an important predictor of post-transplant AH [65]. According to the ISHLT registry, the prevalence of AH among HTx patients is 50%–90% and is associated with an increased cardiovascular morbidity and mortality [66]. There are several con-tributors to the development of AH after HTx such as the use of CNIs and corticosteroids for immunosuppression, the negative effect of cardiac denervation and nephropathy, which addition-ally activates the renin—angiotensin system, hereby increasing the intravascular fluid volume and peripheral resistance [56, 67, 68]. Activation of the sympathetic nervous system, vasoconstric-tion affecting endothelin-1, reduction in circulatory nitric oxide and prostaglandin concentrations, are the main mechanisms of CNI-induced AH [69]. Polymorphisms resulting in CYP3A5 loss of function may also significantly influence drug metabolism and exposure, and lead to higher incidence of CNI-related nephrotoxicity [70].

According to the 2018 European Society of Cardiol-ogy/European Society of Hypertension (ESC/ESH) guidelines and 2023 ESH guidelines for the management of AH, a diagnosis of AH is established when office systolic BP is *>* 140 mmHg and/or diastolic BP is *>* 90 mmHg [68, 71]. According to the SPRINT clinical trial, among patients who were at increased cardiovascular risk, targeting a systolic BP *<* 120 mmHg resulted in lower rates of major adverse cardiovascular events and lower all-cause mortal-ity [72]. However, there are no data about specific BP levels that should be maintained in the HTx population other than referring to the recommended BP levels to non-HTx recipient population levels and taking into account patients’ ethnicity and the other recipients’ comorbidities (i.e., chronic kidney disease [CKD], etc.). Throughout the management of AH, ambulatory BP monitoring is warranted, in particular when in doubt regarding the accurate diagnosis or the adequacy of BP control.

Once AH is diagnosed as per respective guidelines, interventions need to be implemented as the benefits in HTx patients are similar to those in patients with AH at large [71]. Efforts to achieve the lowest possible effective CNI serum level (tacrolimus, cyclosporine) and, if possible, to discontinue corticosteroids by the end of the first year post-transplant are warranted [56]. Finally, antihypertensive therapies should be initiated if needed. Drug selection is empiric and depends on BP responses [56]. Adequate BP control with a calcium channel blockers (CCB) or angiotensin-converting enzyme (ACE) inhibitor is warranted to avoid CKD [56]. Data show that the majority of HTx recipients required only a single antihypertensive drug, and CCB are primarily used [73]. The reason for such choice is their neutral effect on cardiac and renal function and minimal interaction with IS drugs [74], whereas ACE inhibitors and angiotensin receptor blockers (ARBs) may be beneficial among diabetic recipients, and a two-drug regimen can include both CCB and ACE inhibitor/ARB [56, 75, 76]. Another concern about using ACE inhibitors and ARBs, especially in patients with CKD, is that the serum creatinine level tends to rise when starting these drugs, although several studies have shown that an acute rise in creatinine may demonstrate that the drug is actually protecting the kidney. This phenomenon was described as “pre-renal success,” proposing that the decline in glomerular filtration rate is haemodynamic, secondary to a fall in intraglomerular pressure as a result of efferent vasodilatation, and therefore should not be reversed. Caution is advised when initiating ACE inhibitor/ARB therapy in these high-risk groups as well as in patients with potassium levels *>*5.0 mmol/L at baseline, at high risk of pre-renal acute kidney injury, with known renal insufficiency, and with previous deterioration in renal function on these medications [77].

In conclusion, a first-line antihypertensive therapy in heart recipients without diabetes or with advanced CKD (stages IV–V) or at high risk of acute kidney injury development is CCB. Otherwise, HTx patients with diabetes or CKD (stages I–III) will benefit from ACE inhibitor/ARB prescription. If first-line antihypertensive medications (CCB or ACE inhibitors/ARBs) are not effective, then instead of its dosage up-titration either CCB or ACE inhibitors/ARBs should be added.

### Dyslipidaemia

Dyslipidaemia is frequent in HTx recipients, not just because of background history and comorbidities, but also because IS therapy has a negative impact on the lipid profile [78]. Dyslipidaemia is associated with CAV and cardiovascular events [79].

Statins have been shown to reduce CAV and improve long-term outcomes and should be started during the very first weeks in all HTx patients, adults and children, regardless of cholesterol levels [56, 80].

The adagio “the lower the better” also applies to the HTx population, aiming at a minimal low-density lipoprotein cholesterol of ≤70 mg/dL (1.8 mmol/L). Initial statin doses should be lower than those recommended for hyperlipidaemia, due to concern for pharmacological interactions with CNIs, and up-titration should be careful, especially in patients on cyclosporine [79].

Interactions between statins and CNIs are well documented, but recent data, though limited, suggest that the combination of tacrolimus and statins may be safe. Retrospective studies sup-port the safety of high-dose statin therapy in patients treated with tacrolimus-based immunosuppression, with greater statin dose to be associated with a reduction in adverse cardiovascular out-comes after HTx [81, 82]. Pravastatin was included in the guidelines due its beneficial effects on cholesterol levels and improved inci-dence of allograft rejection causing haemodynamic compromise, 1-year survival, and the incidence of CAV [56, 83]. Dosage of statins in the HTx population is lower than in general population because of the high risk of myopathy or myositis development [56]. Rec-ommendations for dyslipidaemia medications are presented in Table 1 [56, 84].

**TABLE 1:** Recommendations for the doses of dyslipidaemia tablet medications in the heart transplant population^[56,84]^.

Drug	Doses of dyslipidaemia drugs	Risks of myositis	Interactions between dyslipidaemia drugs and common medications prescribed to heart recipients
Pravastatin (preferred drug)	20–40 mg	Myositis (lower)	Major interactions with cyclosporine – is not generally recommended! Preference should be for the combination of fluvastatin and cyclosporine
Simvastatin	5–20 mg	Myositis (higher)	
	>20 not recommended		
Atorvastatin	5–20 mg	Myositis (higher)	Moderate interactions with tacrolimus, possible use under the control of CO targets of tacrolimus
Fluvastatin (preferred drug)	40–80 mg	Myositis (lower)	
Lovastatin	20 mg	Myositis (higher)	
Rosuvastatin (limited in case of chronic kidney disease)	5–20 mg	Myositis	The combination of warfarin with rosuvastatin/lovastatin—moderate interactions, not recommended
Ezetimibe	10 mg	—	Cyclosporine, anticoagulants
Fenofibrate	145 mg	Myositis (lower)	Cyclosporine, anticoagulants

Ezetimibe may be used as an alternative, or additionally to a maximally tolerated dose of statin in patients who are poorly tolerant to statins, or those with significant dyslipidaemia despite maximal dose statin treatment and dietary advice.

Recently, a retrospective study showed the effectiveness of proprotein convertase subtilisin/kexin type 9 (PCSK9) inhibitors in lowering cholesterol levels in HTx patients, hereby stabilizing coronary intimal hyperplasia [85]. For the specific management of hypertriglyceridaemia, caution is warranted when using fibrates in.

### Thromboembolic Complications

Venous thromboembolism (VTE), including deep venous thrombo-sis and pulmonary embolism, is a frequent complication after HTx, being six times more common among HTx recipients than among the general population [86, 87]. The highest risk is during the first post-operative year [86].

“Classic” risk factors for VTE, such as recent hospitalization, being older, obese, previous history of VTE or having renal dysfunction, all increase the risk of VTE after HTx [86]. However, specific risks exist in HTx recipients: indeed, the use of mTOR inhibitors has been associated with a significant increased risk for VTE, even when controlling for other risk factors [88, 89]. Although strong evidence is still lacking, in order to reduce the risk of VTE after HTx [36], weight loss should be recommended in obese patients [87]. The use of mTOR inhibitors after HTx should take into account the risk of VTE over time [88, 89]. However, indications for mTOR initiation after HTx is based on patients’ comorbidities and/or post-transplant complications. Developed complications, that is, CAV, CKD, malignancy, neurological complications and so forth, outweigh the risk of VTE when conversion to mTOR should be considered. A more aggressive approach to thromboprophylaxis is advised in order to minimize thromboembolic complications [86–89], taking into consideration the potential interactions with other drugs administered to HTx patients.

### Osteoporosis

Osteoporosis represents a serious complication for HTx recip-ients, mainly resulting in vertebral compression fractures, with a prevalence ranging from 14% to 40% [90]. Bone mineral density.

(BMD) is rapidly reduced during the first 6–12 months after HTx [91]. The most important risk factors are pre-transplant bone disease [92] and post-transplant IS therapy, in particular glucocorticoids and CNI use [92]. Other factors such as aging, tobacco use, alcohol con-sumption, nutritional deficiencies, immobility, and hypogonadism can further enhance this risk [93]. After an initial increase of bone resorption markers associated with a decrease of bone formation markers, there is later attenuation in the rate of BMD loss after the first year post-HTx, reflecting a partial normalization of bone for-mation/degradation markers in the later post-HTx period [94]. Sup-plementation with calcium and vitamin D alone may not prevent significant BMD loss [94], while a recent meta-analysis [95] concluded that bisphosphonates effectively reduced the loss of vertebral BMD in early stage after HTx. Resistance ET can provide an additional osteogenic stimulus, and the combination of resistance ET with bisphosphonates is more efficacious than bisphosphonates alone in restoring BMD [11].

### Post-Transplant Diabetes Mellitus

Post-transplant diabetes mellitus is a common complication after HTx, occurring in 20%–30% of HTx recipients [96, 97]. Pre-existing diabetes risk, IS agents and infections are major contributors to the development of PTDM [54]. However, the few available retrospective studies in HTx show that PTDM may increase the likelihood of rejection, AH, renal failure and infection rates [98–100].

Screening for PTDM should be performed once stable doses of immunosuppression are reached. Glycated haemoglobin should not be used as a sole screening measure in the first year after HTx as it could underestimate PTDM due to blood loss and greater red blood cell turnover.

Once diagnosed, PTDM will require action to achieve gen-eral treatment targets of diabetes management. On top of health lifestyle measures that include PA, oral hypoglycaemic medications alone or in combination with insulin therapy are warranted. The new oral antidiabetics such as sodium—glucose cotransporter 2 (SGLT2) inhibitors and glucagon-like peptide-1 receptor agonists (GLP1-RA) seem to be safe according to the first studies and are promising, especially in the setting of transplant vasculopa-thy (GLP1-RA) or renal failure (SGLT2 inhibitors) after HTx [101, 102]. However, after HTx there are no indications to continue SGLT2 inhibitor therapy if it was prescribed prior to surgery. Data on using SGLT2 inhibitors in HTx recipients are limited. According to recent data, SGLT2 inhibitors did not deteriorate heart transplant function and were efficient in diabetes management but during the first year after HTx its possible adverse effects (i.e., urinary tract infection, dyslipidaemia and increased urination) outweighed expected bene-fits. Larger studies of GLP1-RA and SGLT2 inhibitors are needed in patients after solid organ transplantation [102]. There is no evidence that the risk of PTDM can be decreased (e.g., by use of cyclosporine instead of tacrolimus or steroid avoidance/withdrawal), without increasing the risk of rejection.

### Vaccination

The timing of vaccination appears to be critical to optimize responses. The first 6 months after transplantation are associ-ated with the poorest immune response because the patients are usually receiving the highest doses of immunosuppression [103]. No reduction or discontinuation of immunosuppression should be considered because of vaccination in transplant individuals. In the post-transplant setting, inactivated vaccines can be safely administered starting at 3–6 months post-transplantation except for influenza vaccine which can be given as early as 1 month post-transplantation [104].

COVID-19 vaccination is advised in all patients with CHF and a compromised immune system, including patients following HTx receiving IS therapy. They are however unlikely to generate a completely protective immune response after COVID-19 vaccina-tion, and therefore need additional personal measures including facemask wearing and social distancing for added protection [105]. According to the ISHLT guidance, both mRNA (Pfizer/BioNTech, Moderna) and non-replicating viral vector (Sputnik V, AstraZeneca, Johnson &amp; Johnson) vaccination can be used after HTx [106]. The additional dose of the vaccine beyond the standard scheme may increase the efficacy of vaccination in these patients [106].

## Factors Affecting Exercise Capacity

### Effect of Cardiac de- and Reinnervation on Exercise Capacity

Heart transplantation leads to total (i.e., both sympathetic and parasympathetic) denervation of the transplanted heart. Initially this results in increased resting heart rate (HR), due to the loss of vagal tone on the sinoatrial node, and a blunted HR increase during exercise [1, 11]. In denervated patients, no increase in HR is observed the first few minutes after exercise initiation. This phase is followed by a rise in HR rate presumably due to circulating catecholamines, but the increase is reduced compared to healthy subjects [11]. The decreased HR reserve (HRR) after HTx has generally been assigned an important role in the well-described exercise limitation observed in most patients; however, there is no consensus about the relative importance of HRR compared with contractile and diastolic reserve or peripheral factors [107].

Cardiac reinnervation occurs in a subgroup of HTx recipients. Both sympathetic and some degree of parasympathetic reinnerva-tion has been demonstrated, although the latter is much less well established [108]. The degree of reinnervation depends on several factors, including time from transplantation, age of recipient and donor as well frequency of rejection episodes [13].

Reinnervation after HTx is associated with a higher peak HR and HRR as well as a greater exercise tolerance [109]. High-intensity interval training (HIT) after HTx can increase peak HR and HRR, but whether this indicates an effect of exercise on reinnervation is not known. HIT clearly improves VO_2peak_ but the improvement in VO_2peak_ and HRR is not correlated, hence the improvement in VO_2peak_ is largely related to peripheral (muscular) adaptation [110].

### Post-Heart Transplant Arrhythmias During Exercise

Transplantation leads to a denervation of the sympathetic and parasympathetic nerve fibers, that significantly restricts HR vari-ability [111]. Moreover, reactive tachycardia in response to underlying physiologic distress (e.g., pain, hypovolaemia, intense effort, etc.) may be blunted since the response of the grafted tissue to intrin-sic catecholamines is variable [112]. The transplanted heart shows a delayed chronotropic response to exercise due to a reliance on cir-culating catecholamines [37]. Denervation gradually leads to an emp-tying of the catecholamine stores in the myocardium, meaning that the transplanted heart is then reliant on the stimulation of circu-lating catecholamines. Overall, the catecholamine receptors display an increased sensitivity. In some cases, this can lead to an increased incidence of cardiac arrhythmia, whereby for decades therapy with beta-blockers was not considered the preferred option because it has the capacity to significantly reduce exercise tolerance [113, 114]. However, in recent years some evidence has shown that after HTx beta-blockers are useful and effective in the treatment of cardiac arrhythmias, left ventricular systolic dysfunction and AH and in long term after HTx can lead to a HR reduction [114, 115]. Most sudden cardiac deaths occurring in HTx recipients are related to trans-plant coronary artery disease or CAV [113]. Most cases of sudden cardiac death have asystolic presentation, and ventricular fibrilla-tion occurs only in 10% of the patients with moderately depressed or preserved left ventricular ejection fraction [116]. According to current evidence, ET appears to be a safe intervention in HTx recipients and it has not been related to an increased risk of devel-oping cardiac arrhythmias [9]. However, after the first detection of post-transplantation cardiac arrhythmia, HTx recipients should be examined with transthoracic echocardiography, 24 h electrocar-diogram (ECG) monitoring, endomyocardial biopsy and coronary angiography to rule out allograft rejection and CAV [55, 113]. Transplant recipients undergoing exercise therapy should begin in a supervised setting with continuous ECG monitoring. More than one-third of subjects exhibit a partial normalization of HR response to exercise from6 months to 1 year after surgery [39].

### Atherosclerosis and Cardiac Allograft Vasculopathy

Cardiac allograft vasculopathy is one of the leading causes of increased morbidity and mortality (which occurs in 32% of the patients at 5–10-year follow-up) in HTx recipients [117, 118]. CAV is multifactorial and is caused by immunologic mechanisms, and stim-ulated by non-immunologic factors leading to persistent endothe-lial injury [119, 120]. Intimal hyperplasia progresses towards coronary obstruction, which impairs perfusion up to the point of graft failure [118].

Prediction or early clinical diagnosis of CAV is difficult. Owing to the denervation of the transplanted heart, patients with CAV do not usually experience chest pain, but typically are asymptomatic until they present with sudden death or congestive heart failure [117]. Surveillance against CAV is currently performed by periodic coronary angiography. Intracoronary imaging with intravascular ultrasound or optical coherence tomography is useful in serial assessment of disease progression [121]. However, these techniques are invasive and expensive. Therefore, promising non-invasive modalities for an early detection of CAV like computed tomog-raphy angiography with coronary fractional flow reserve, car-diac magnetic resonance imaging and positron emission tomog-raphy assessment are under investigation [122]. Future studies will confirm whether these techniques may allow stratifying high-risk HTx patients for CAV development reliably and non-invasively, and may therefore substitute the current gold standard invasive intra-coronary imaging.

Conversion from a CNI- to a sirolimus-based IS regimen attenu-ates CAV progression and results in a positive remodelling effect on the coronary artery wall. Beneficial volumetric changes occur with conversion to sirolimus resulting in reduced rates of CAV-related events and improved late survival, in which the greatest benefits are achieved when patients are converted early (within 6 months to 2 years) following HTx [123]. On the other hand, total cholesterol and triglycerides seem to increase significantly in the sirolimus con-verters, although the dyslipidaemia associated with sirolimus does not translate into higher rates of cardiac events [123]. Actually, com-pared with continued CNI therapy, sirolimus attenuates plaque progression in recipients with early conversion, but contributes to increases in necrotic core and dense calcium volume in those with late conversion [124].

### Post-Heart Transplant Nutrition

Optimal nutrition is an important part of the management for the heart transplanted patient. Weight control and lipid-lowering diet are recommended for all heart transplanted patients, irrespective of their sex, age, or aetiology of CHF [56, 78, 125]. Excess body weight worsens lipid profile and glycaemic control, and increases the risk of atherosclerosis and AH. CNIs (tacrolimus, cyclosporine) are well-known potent IS agents affecting electrolyte levels with known drug interactions. Grapefruits, pomelo, ginger, St. John’s wort and turmeric juice should be avoided after HTx due to drug inter-actions and changes in immunosuppression blood levels [126, 127]. In addition, tacrolimus affects potassium and magnesium levels and mTOR inhibitors cause elevation in blood lipid levels. Appropri-ate nutrition considering the blood electrolyte and lipid levels should be tailored in every heart transplanted patient and those with diabetes should be counselled regarding weight control and low-glycaemic diet [55, 125].

### Skeletal Muscle Abnormalities

Skeletal muscle abnormalities are a major factor limiting exercise capacity in patients with CHF that are partially reversed by ET [128], however this has been less well studied in patients post-HTx. Following HTx, patients demonstrate numerous skeletal muscle deficits that include loss of mass and function alongside evi-dence of mitochondrial abnormalities, a shift towards Type II more-fatiguable glycolytic fibres, and reduced fibre capillarity [129]. Skeletal muscle deficits following HTx can persist for months and are closely correlated to measures of exercise capacity [130], which provides a fundamental explanation for why many HTx patients are unable to retain normal exercise/functional capacity promptly. For-tunately, traditional endurance or strength ET regimes performed over 3–6 months can normalize at least some of the skeletal mus-cle deficits post-HTx, by increasing muscle mass and strength in line with greater mitochondrial function/morphology and Type I fatigue-resistant oxidative fibres, although low fibre capillarity ratio persists [129]. Collectively, therefore, skeletal muscle is a key periph-eral organ post-HTx that limits exercise/functional capacity but is modifiable by sustained ET.

### The Impact of Frailty on Post-heart Transplant Follow-Up

Multidimensional frailty (including the physical, psycho-cognitive, social, nutritional domain) is highly prevalent in HTx patients [131, 132]. Among all the components of multidimensional frailty, some can be reversible (treatable) while others are irreversible (requiring sup-portive care) [132]. The identification of the major components of frailty, if present, and the role exerted by each domain are the pil-lars to prioritize interventions within a tailored plan of care after HTx [133]. Frailty which develops during the waiting list period, is often the basis to plan rehabilitative interventions. Moreover, it has been demonstrated that frailty within 6 months before HTx is associated with increased mortality and prolonged hospitaliza-tion after HTx [134, 135]. Therefore, the identification of a common language to evaluate frailty among CHF specialists is mandatory. The need for a common language to manage CHF and transplanted patients has been proposed in recommendations from the ESC, the American Heart Association (AHA), and the Society for Geri-atric Cardiology (SGC) and these emphasize the importance of awareness of the frailty syndrome in the treatment of patients with CHF [29]. Recently, a quick tool to identify multidimensional frailty was proposed to reduce the time spent in frailty evalua-tion [136–138]. AGILE is a 10-item tool evaluating mental, physical, socioeconomic and nutritional domains (Table 2) with the ability to predict mortality, disability and hospitalization, which is especially useful in care settings that require reliable assessment instruments with short administration time [139].

**TABLE 2:** The 10 items of the AGILE tool with relative scoring divided by domain of frailty (physical, mental, nutritional, and socioeconomic).

No.	Item	Score	Frailty domain
1	Feel everything is an effort	Yes – 1, No – 0	Physical
2	Help up/down stairs	Yes – 1, No – 0	
3	Grip strength^a^	Yes – 1, No – 0	
4	Temporal orientation deficit^b^	Yes – 1, No – 0	Mental
5	Delayed recall deficit^c^	Yes – 1, No – 0	
6	Feel depressed	Yes – 1, No – 0	
7	Weight loss over 4.5 kg in the last year	Yes – 1, No – 0	Nutritional
8	Help in eating	Yes – 1, No – 0	
9	Financial help from family members	Yes – 0, No – 1	Socioeconomic
10	Physical help from family members	Yes – 0, No – 1	

^a^ ≤30 kg in men, ≤20 kg in women at hand-held dynamometer.

^b^ The subject does not refer the exact date (day/month/year).

^c^ The words “bread-house-cat” are referred to the subject at the beginning of the questionnaire and then asked to the subject at this time of the questionnaire.

### Associations Between Exercise and the Immune Landscape

Regular ET modulates the immune landscape, affecting both num-bers of immune cell subtypes as well as their activation state and responsiveness to activation [140–143]. Hence, ET might in principle support shaping the host’s immunity in HTx in a way to improve graft survival as well as pathogen defence in addition to its role in aiding physical recovery. The exercise-induced effects on immunity, however, need to be viewed in context with the IS regimen used for each patient.

A better understanding of the role of individual leucocyte populations and their modulation by exercise under the various IS regimens is needed to develop effective prevention and treatment strategies against acute and chronic graft-versus-host disease [144, 145]. High-intensity interval training is more effective for enhancing aerobic fitness of sedentary males by increasing their pulmonary ventilatory and cardiac haemodynamic responses to exercise than moderate intensity-continuous ET and these experimental findings facilitate the identification of effective ET regimens to increase aerobic capacity and minimize immune death under conditions of hypoxia [146].

Endothelial cells serve as facultative antigen presenting cells and thereby take on a crucial role in graft rejection. Indeed, ET has been shown to preserve endothelial function in HTx recipients [130, 147, 148]. The maintenance or improvement of endothelial integrity and quiescence may therefore contribute to the beneficial role of endurance exercise in HTx rehabilitation.

Finally, exercise might have a role in counteracting the side-effects of immunosuppression, which include increases in plasma glucose, lipids and/or BP and skeletal muscle dysfunction in addition to an increased risk of infections. There is individual evidence that ET might alleviate these to some degree [11, 12], albeit systematic studies are lacking and relevant changes in the immune landscape in this context have not been systematically investigated.

## Rehabilitation After Heart Transplantation

### Early- and Long-Term Cardiac Rehabilitation

Early-term rehabilitation after HTx could be delivered in the usual phases I and II (Table 3) of CR. During the in-hospital phase, early mobilization—particularly in phase I but also in phase II CR—can be initiated as soon as haemodynamic reestablishment and weaning from post-transplant intravenous drugs occurs [2]. Phase I refers to postoperative early mobilization, patient education and promotion of adherence towards following phase II activities, being the last couple also part of an eventual prehabilitation course (i.e., before HTx). Phase II CR (initiated before discharge and followed by post-discharge pre-exercise programme) in these patients usually starts with an in-patient programme (due to the complexity of intervention and related needs of strict observation), directly followed by outpatient activities [2]. The average duration of hospital stay after patients underwent HTx with uncomplicated early-term follow-up and before the discharge from the hospital is 2–3 weeks [149–151]. Considering the time difference of recipients’ post-transplant stay at the hospital the duration of the phase 1 can be different, the same as when the phase II can be initiated which is more than 2–3 weeks after HTx. Time windows of residential and ambulatory CR is about 3–4 weeks and 2–3 months, respectively, varying with local policies. At discharge, heart recipients should be able to walk on a level surface for a period of 40–60 min at speeds of 80–100 m/min, 4–5 times a week [2].

**TABLE 3:** Phases of cardiac rehabilitation.

Phase I	In-hospital patient period
Phase II	Post-discharge pre-exercise period
Phase III	Exercise and education programme
Phase IV	Maintenance

Long-term multidisciplinary CR is very important after HTx [152] and includes phases III (exercise and education programme) that is followed by phase IV (maintenance) [2]. Only half of HTx recipients participate in CR programmes, and those who do have a lower 1-year readmission risk [153]. All heart transplant team healthcare specialists should participate in the post-transplant rehabilitation programmes.

Despite having a new heart, these patients may suffer from exercise limitation in relation with muscle abnormalities resulting from their previous CHF and comorbidities, anti-rejections thera-pies, corticoid myopathy and from deconditioning due to muscle pain or fatigue. HTx recipients are therefore often severely decon-ditioned [154]. Residual peripheral vasodilatory limitation may persist after surgery [9]. At least in the first months, exercise capacity is also limited by chronotropic incompetence. Therefore, improving peripheral (muscular) performance, mediated by amelioration of microvascular and/or skeletal muscular metabolic function, is key for achieving a sustained improvement of exercise capacity. In fact, various studies have shown that regular physical ET is effective in improving exercise capacity and HRQoL in these patients [9].

Specific core components of CR after HTx—to be integrated by common components of CR in cardiovascular patients [2] —are summarized in Table 4.

**TABLE 4:** Core components of cardiac rehabilitation after heart transplantation^[2,75]^.

Component	Issue
Patient assessment and self-assessment	• Clinical stabilization
	• Wound healing
	• Risk of acute rejection
	• Exercise tolerance
	• Personal and dental hygiene, risk of communicable diseases
	• Avoidance of environmental risks
PA counselling	• Advice for PA as a way for return to functional lifestyle with good quality of life
	• Consideration of both AET and RST training
	• Self-monitoring of exercise intensity more relied on perceived exertion than HR range
Structured exercise training	• Phase I: respiratory physiotherapy, active and systematic mobilization of the upper and lower limbs
	• Phase II: AET in the second or third week after HTx, RST after 6–8 weeks
	• AET intensity: <50% VO_2peak_/W_peak_ (or 10% below AT)
	• RST intensity: 40–70% 1-RM
Diet/nutritional counselling	• Dietary choices, particularly concerning foods to be avoided
Weight control management	• Avoidance of overweight to balance the side-effects of immunosuppressants and to limit cardiovascular risk factors for CAV
	• Consider weight loss for obese patients
Lipid management	• Pharmacological and non-pharmacological treatment of hyperlipidaemia as a risk factor for CAV
	• Choice of statin with regard to interaction with cyclosporine and other immunosuppressants
Blood pressure monitoring	• Target blood pressure: ≤140/90 mmHg
	• Relationship between hypertension and immunosuppression/heart denervation
	• Consideration of CCB and ACE inhibitors/ARB as first choice
	• Beta-blockers are not the first choice of therapy to manage AH due to they can delay chronotropic response but they can be added to the combination of antihypertensive drugs if CCB with/or ACE inhibitors/ARB are not efficient to reach the required blood pressure
Smoking	• Smoking cessation
	• Avoidance of post-HTx smoking resumption
Psychosocial management	• Clear advice on life after HTx
	• Structured support and intervention for psychosocial risk factors

1-RM, one repetition maximum; ACE, angiotensin-converting enzyme; AET, aerobic endurance training; AH, arterial hypertension; ARB, angiotensin receptor blocker; AT, anaerobic threshold; CAV, cardiac allograft vasculopathy; CCB, calcium channel blocker; HR, heart rate; HTx, heart transplantation; PA, physical activity; RST, resistance/strength training; VO_2peak_, peak oxygen consumption; W_peak_, peak workload.

For exercise rehabilitation, one may use any of the usual of training methods in these patients (continuous aerobic or interval training, resistance training, inspiratory muscle training). One should, however, not rely on the HR response for train-ing prescription and assessment in case of denervation-related abnormal chronotropic response. Finally, it has been shown that these patients need long-term supervised programmes because short-term or home-based programmes without proper remote guidance may be less effective than in other patients [155].

### Exercise After Heart Transplantation: Practical Implementation

After HTx, ET and PA implementation play a key role in the rehabilitation programme, and need a detailed consideration of the following steps: risk stratification assessment, shared-decision making, data monitoring, exercise modalities adjustment, and consideration of any other issues that arise.

#### Risk Stratification Assessment

A symptom-limited CPET prior to any exercise intervention, with monitoring of ECG, ventilatory parameters (i.e., ventilatory VO_2_, CO_2_ production, ventilation), workload, oxygen saturation and BP should be performed [2, 37, 156]. From this the first and second ventilatory threshold can be determined [156]. In addition, a 6-min walking test maybe used to document functional capacity where such CPET facilities are not available. One-repetition maximum (1-RM) testing is advised to assess muscle strength, or in patients who cannot tolerate completing CPET (especially in patients with a difficult postoperative course) [156]. These data can then be used for personalized exercise prescription, to maximize the safety of exercise and decide on the appropriate setting for subsequent ET (e.g., supervised, hospital, gym, home-based).

#### Shared Decision-Making

When prescribing exercise/PA, it is necessary to consider patient preferences to maximize adherence and to optimize the intervention goals [2].

#### Data Monitoring

In particular during the first few weeks of a newly started exercise/PA intervention, monitoring BP and HR ahead of exercise, HR, oxygen saturation, and Borg rating of perceived exertion dur-ing endurance ET, OMNI-RES during strength training, BP and HR after exercise are recommended [2, 156]. In some patients, continuous ECG monitoring can be beneficial (based on ischaemic, arrhythmic and clinical status).

#### Exercise Modality Adjustment

The HTx patient generally has—at least initially—a delayed HR response due to cardiac denervation. As a result, a warm-up period before each session and steady-state aerobic exercise are recom-mended at the beginning. Although HTx patients are expected to start with a low exercise load and muscle strength, (rapid) changes are expected to occur. Functional electrical stimulation is advised in special cases where mobilization is not possible soon after surgery. As a result, regular assessments of physical fitness (endurance capacity and muscle strength) are needed to adjust the duration and/or intensity of the exercise sessions [154]. CPET can be advised after 6 weeks (useful in individualized ET for phases III–IV of CR) in the first 3–6 months of intervention (before the reinnervation and development of HR variability) and then at least once per year to estimate the dynamic of physical capacity, while 6-min walking tests and 1-RM tests can be executed more often (e.g., every 2 weeks) while hospital admission or check-ups in the outpatient department [2, 37, 107, 157, 158]. The end-goal should remain HIT, if tolerated by the patient, with periods of 85%–95% of maximum HR.

#### Other Issues

Exercise performance is also affected by nutritional status, psy-chosocial status (e.g., level of education, economic status, believes about exercise/PA) and/or motivation. Education on exercise/PA is very important, and the motivational stage should be taken into account.

### High-Intensity Interval Training After Heart Transplantation

Up until a study by Haykowsky et al. [158] in 2009, exercise after HTx had, and in many institutions still has, a rather conservative approach consisting mainly of moderate intensity continuous train-ing, mostly due to uncertainty and concerns regarding denervation and consequently chronotropic incompetence and parasympa-thetic impairment. Recent studies demonstrated that HIT was safe and effective in different groups of maintenance recipients (*>*1 year post-HTx).

Since 2009, accumulating evidence has underscored these early findings regarding the safety and efficiency of HIT in HTx recipients. The first meta-analysis of exercise studies in solid organ transplant recipients was published in 2013 [159], which concluded that “ET is a promising but unproven intervention for improving the cardiovas-cular outcomes,” has already been largely replaced by more positive recommendations. A recent European Association of Preventive Cardiology position paper stated that previous restrictions placed on HTx recipients with regard to exercise modalities, and especially HIT, do not seem to rely on evidence-based knowledge [2].

Although more research is still needed in different aspects of the field, newer studies have addressed both long-term effects of HIT [108, 160], possible mechanisms for the “HIT effect” [109], comparison of exercise modalities [110, 157], HIT in *de novo* HTx recipients [111], and demonstrated stronger evidence of reinnervation [161].

In summary, the vast majority of performed studies have proven positive effects of HIT on multiple factors as, for example, VO_2peak_, muscle strength, chronotropic responses, CAV, body composition and HRQoL [9, 37, 38, 159, 162–165]. Although beneficial effects of HIT on HTx recipients seem to differ to some extent from patients with coronary heart disease or heart failure, with more prominent peripheral effects of exercise, rather than central adaptions such as increased stroke volume, there is no doubt that HIT is highly effective in HTx recipients and should be more frequently used. However, individual tailoring and individual considerations are still needed to determine the optimal exercise modality for each specific patient.

### Benefits of Exercise Rehabilitation After Heart Transplantation

**FIGURE 1:**
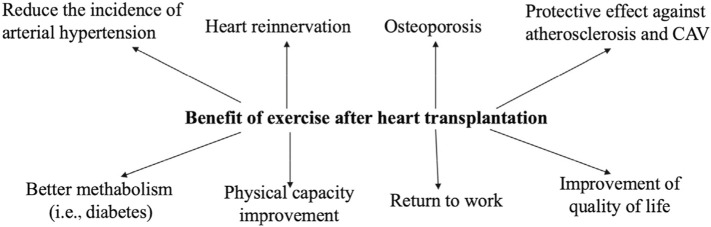
Benefit of exercise after heart transplantation. CAV, cardiac allograft vasculopathy.

There are benefits of post-transplant exercise rehabilitation on reducing post-transplant complications as follows (Figure 1).

#### Arterial Hypertension

Exercise training is widely used for reducing BP in hypertensive subjects. Among studies investigating potential benefits of ET in HTx recipients only few have used BP as primary endpoint; therefore, most of the available data come from studies designed with different endpoints in which resting BP or ambulatory BP were secondary or exploratory variables. A 12-week training programme performed at 69% of VO_2peak_ was effective for reducing both systolic and diastolic ambulatory BP in HTx recipients [166]. Little is known about the type, frequency, or intensity of exercise that provides the greatest benefits on BP of HTx recipients. HIT proved to be more effective than no training [167], and slightly better than continued moderate exercise on reducing systolic BP [10] but it failed to reduce BP in another [40]. It is possible that denervation that occurs during transplantation surgery may reduce the hypotensive response to ET in HTx compared to what has been observed in other populations. In a recent study, a greater reduction of ambulatory BP, as well as a greater increase of maximal VO_2_, were observed in patients with evidence of cardiac reinnervation compared to those without cardiac reinnervation [168].

#### Diabetes

The role of ET in the management of diabetes in HTx recipients has been poorly investigated. A recent meta-analysis, evaluating the effects of exercise on components of metabolic syndrome and involving patients with solid organ transplantation, showed a significant reduction of fasting blood glucose after training [169].

#### Osteoporosis

Strength training, alone or in association with drug therapy, has long been recognized as an effective intervention in counteract-ing bone loss in HTx recipients [170]. Six months of strength training potentiated the effects of alendronate administration on revert-ing glucocorticoid-induced osteoporosis in HTx recipients [11]. In another study strength training combined with the administration of calcitonin was more effective than calcitonin alone in restor-ing BMD in spine to within 5% of pre-transplantation levels within 8 months after HT [171].

#### Heart Reinnervation

The HR response to exercise is one of the most important pre-dictors of exercise capacity in transplant recipients with com-plete chronotropic competence and without relevant transplant vasculopathy or acute allograft rejection [172]. Transplant recipients with evidence of restoration of sympathetic innervation had bet-ter exercise performance compared to denervated recipients, due to a better chronotropic and inotropic response. Overall exercise time was significantly greater in reinnervated patients with a signifi-cantly greater increase of HR above baseline, and peak HR attained during exercise compared with denervated patients. Multiple stud-ies have demonstrated the benefit of ET after HTx by improving VO_2_peak, peak HR, and chronotropic response, and high-intensity, interval-based aerobic exercise has been documented to have supe-rior positive effect compared with moderate exercise [13]. ET was effective to reduce BP, to lead to HR variability and to increase exercise tolerance. However, it was not effective to improve arte-rial stiffness [168, 173]. The fact that the improvement in exercise capac-ity is lost after a few months without training, may suggest that the physiological mechanisms for improvement are primarily peripheral and not through cardiac remodelling [13].

#### Atherosclerosis

Exercise training has a protective effect against the development of CAV. In a murine model, ET reduced the onset of CAV by enhancing endothelial cell regeneration and function in the graft [33]. In humans, Nytrøen et al. [174] reported significantly reduced progress of CAV in HTx recipients undergoing HIT compared with no exercise. Among potential anti-atherosclerotic mechanisms of exercise in HTx recipients is a reduction of the inflammatory response. In a small study, exercise evoked an immediate response in several vascular, angiogenetic and platelet-derived inflammatory mediators in HTx recipients, irrespective of the training intensity [109].

#### Quality of Life

Studies investigating post-transplant HRQoL have clearly demon-strated that HTx recipients have significantly improved HRQoL compared to the pre-transplant stage [37]. This supports previ-ously documented evidence on the association between increased exercise capacity and better HRQoL [37]. A moderate level of exercise and intensity is insufficient to maintain the higher VO_2peak_ that were achieved after the HIT intervention [160]. It was suggested that HIT can reduce the development of anxiety symptoms in the long term, which is a frequent health issue following HTx [160]. The.

HIT group reported significantly less anxiety symptoms, but there were no long-term differences in VO_2peak_, muscular capacity, or CAV between the groups [160]. In addition, pediatric HTx adoles-cents do not meet their required PA recommendations. Despite this, they have low normal exercise capacity and report a normal HRQoL. Efforts to engage adolescents to increase their PA should be encouraged [175]. Young adult transplant patients are to be care-fully evaluated for psychosocial risks to avoid non-compliance and reduced HRQoL in the long term [176].

## Gaps in Knowledge

Life-long follow-up proved its benefit after HTx but it may limit recipients in their socialization and may deteriorate their men-tal health. So future research should focus on incorporating telemedicine, remote consultations and developing digital platform. In addition, CR programmes should be initiated early after HTx and then should be life-long continued. And future perspectives are to organize and to implement CR programmes in the long term as a part of outpatient follow-up. Future projects should provide the particular exercise recommendations for HTx individuals based on their condition and time after surgery. Moreover, further research is needed to establish long-term impacts of rehabilitation and ET on cardiovascular disease incidence and progression.

## Conclusion, Including Open Questions and Future Research

The number of HTx patients increases and it is important to initiate prevention and multidisciplinary rehabilitation from the beginning after surgery and to continue them after discharge. All heart trans-plant team members have their role and need to participate in transplant recipients’ prevention and rehabilitation programmes. After HTx prevention can be defined as a comprehensive set of measures, aiming to reduce the recurrence or development of car-diovascular disease and to improve long-term prognosis. Despite the profound benefits of receiving a heart transplant, recipients need continual psychosocial as well as medical support, based on the understanding of the many complex challenges that confront them. Life-long participation in CR programmes has been shown to improve symptoms and allograft function in the long term. There is a wide range of risk factors (modifiable/non-modifiable) that should be addressed after transplantation and taking them into account may reduce the number of cardiovascular complications and improve recipients’ prognosis.
